# Mindfulness-based stress reduction teachers, practice characteristics, cancer incidence, and health: a nationwide ecological description

**DOI:** 10.1186/s12906-015-0545-3

**Published:** 2015-02-14

**Authors:** Sara Wagner Robb, Kelsey Benson, Lauren Middleton, Christine Meyers, James R Hébert

**Affiliations:** College of Public Health, Department of Epidemiology and Biostatistics, University of Georgia, 101 Buck Road, Health Sciences Campus, B.S. Miller Hall, Athens, GA USA; Cancer Prevention and Control Program and Department of Epidemiology & Biostatistics, Arnold School of Public Health, University of South Carolina, Columbia, SC USA

**Keywords:** MBSR, Meditation, Health, Mental health, Stress, Cancer

## Abstract

**Background:**

Studies have demonstrated the potential of the Mindfulness-Based Stress Reduction (MBSR) program to improve the condition of individuals with health outcomes such as hypertension, diabetes, and chronic pain; improve psychological well-being; reduce stress levels; and increase survival among cancer patients. To date, only one study has focused on the effect of long-term meditation on stress, showing a positive protective relationship. However, the relationship between meditation and cancer incidence remains unexplored. The objective of this study was to describe the state-level relationship between MBSR instructors and their practices and county-level health outcomes, including cancer incidence, in the United States.

**Methods:**

This ecologic study was performed using geospatial mapping and descriptive epidemiology of statewide MBSR characteristics and overall health, mental health state rankings, and age-adjusted cancer incidence rates.

**Results:**

Weak to moderate state-level correlations between meditation characteristics and colorectal and cervical cancer incidence were detected, with states with more meditation (e.g., more MBSR teachers per population) correlated with a decreased cancer incidence. A negative correlation was detected between lung & bronchus cancer and years teaching MBSR only. Moderate positive correlations were detected between Hodgkin’s Lymphoma and female breast cancer in relation to all meditation characteristics. Statistically significant correlations with moderate coefficients were detected for overall health ranks and all meditation characteristics, most strongly for total number of years teaching MBSR and total number of years of general meditation practice.

**Conclusions:**

Our analyses might suggest that a relationship exists between the total number of MBSR teachers per state and the total number of years of general meditation practice per state, and colorectal and cervical cancer incidence. Positive correlations were observed with overall health rankings. Despite this study’s limitations, its findings could serve to generate hypotheses and to inform and motivate a new focus on meditation and stress reduction in relation to cancer incidence, with specific relevance to colorectal and cervical cancer.

**Electronic supplementary material:**

The online version of this article (doi:10.1186/s12906-015-0545-3) contains supplementary material, which is available to authorized users.

## Background

Stress, which nearly 20% of American’s experience at an ‘extreme’ level [1], plays an important role in mental health outcomes and negatively impacts human physiology. Psychosocial stress contributes to adverse mental and physical health effects including anxiety, depression, inflammation, increased blood pressure, rapid breathing, and the increased release of cortisol and epinephrine, all of which may play an important role in chronic disease development (e.g., cardiovascular disease, cancer) [2,3]. Chronic stress may be indirectly related to cancer incidence through related behavioral changes and increases in risky health behaviors such as poor diet, increases in alcohol consumption, and decreased amounts of physical activity.

In addition to its effect on health behaviors and quality of life [4,5], chronic stress activates the hypothalamic-pituitary axis (HPA) and autonomic nervous systems, which results in alterations in neuroendocrine regulation (e.g., prolactin, melatonin) and the immune response (e.g., cell signaling, cytokine levels) [6]. Experiencing chronic stress exacerbates these effects and may be related to adverse health outcomes, including cancer via immune dysregulation [7,8], increased inflammation [9], increased cortisol [10], and altered melatonin levels [11]. Chronic stress has been shown to promote tumor growth and cancer progression in laboratory studies [12,13].

Despite this knowledge, the current health care system provides limited stress management and disease prevention care [1]. Exploration of alternative methods for stress management that can be implemented affordably and effectively without a physician’s care or supervision, such as meditation, should be a priority. As such, stress reduction through long-term meditation practice in particular, may assist in preventing chronic diseases, including cancer [4,11,14,15].

In recent years, the ability of meditation and other mind-body techniques to reduce stress and improve health outcomes in short-term intervention-based settings has received considerable attention [5,16-19]. The Mindfulness-Based Stress Reduction (MBSR) program is a standardized eight-week course developed by Jon Kabat-Zinn that focuses on developing mindfulness through meditation in both formal and informal settings [20]. Observational and experimental studies have demonstrated the potential of MBSR to improve the condition of individuals with various health outcomes such as hypertension, diabetes, and chronic pain [21,22]; improve psychological well-being [18,23]; reduce stress levels [21,24]; and increase survival among cancer patients [25]. However, the existing literature focuses on this or similar meditation-based programs as intervention techniques for reducing stress over short periods of acute stress, primarily among individuals with specific, acute medical complaints [11,15]. To our knowledge, only one study [26] has examined the impact of long-term implementation of MBSR or meditation practices on stress reduction. Brand et al. assessed salivary cortisol levels in a group of 20 meditators, nine of whom were classified as long-term meditators (average experience: 264 months) [26]. Their results indicated a protective relationship between cortisol, a major stress hormone, and duration of meditation experience [26]. Finally, no studies have explored the relationship between long-term meditation with cancer prevention.

A potential relationship between long-term meditation and improved overall health, mental health, and stress reduction remains virtually unexplored. The objective of this paper is to describe, for the first time, the ecological relationship between MBSR teachers and their practices and overall health, mental health, and cancer incidence in the United States. We accomplished this goal through geospatial mapping and descriptive epidemiology of statewide MBSR characteristics and overall health, mental health state rankings, and age-adjusted cancer incidence rates. This initial descriptive study marks a first step in an important and novel research area of the potential relationship between long-term meditation and mental health, stress, and cancer outcomes.

## Methods

Teachers of the MBSR program receive training and certification through the Oasis Institute for Mindfulness-Based Professional Education and Training Center (http://www.umassmed.edu/cfm/stress/index.aspx). A publicly available database on MBSR teachers, in various stages of training, is available through this website and was used to compile the following state-level variables for analysis: total number of MBSR teachers, hours of MBSR taught, years of MBSR taught, and years of meditation practice by MBSR teachers. Population data for each state for the year 2010 were obtained through the US Census [27]. This allowed the calculation of standardized meditation variables per unit population for each state.

Age-adjusted cancer incidence rates for 2007–2011 were obtained from the National Program of Cancer Registries WONDER on-line database [28] for the following cancer sites: colorectal, female breast, prostate, lung & bronchus, melanoma, liver, Hodgkin’s lymphoma, non-Hodgkin’s lymphoma, and cervical cancer. The majority of these cancer sites were chosen a priori because of their potential relationship with stress and its biological consequences on the immune system and inflammatory processes [7-9]. Female breast, prostate, colorectal, and lung & bronchus cancer were included because they are the most commonly diagnosed cancer sites in the United States. Cancer incidence data for 49 states and the District of Columbia were included; data for Nevada were unavailable because the cancer registry did not meet publication criteria for the years under consideration and was excluded from all analyses.

Overall Health Rank and Mental Health Rank data were obtained from America’s Health Rankings (http://americashealthrankings.org), with lower ranks indicating a better overall health status. The Overall Health Rank for each state is a weighted sum of the number of standard deviations each core measure (e.g., physical ability, social support, premature death, mental health days, physical inactivity, and poverty) is from the national average. Mental health data are based on the number of days in the previous 30 days that a person indicated their life activities were limited due to mental health difficulties, based on self-report of CDC’s Behavioral Risk Factor Surveillance System (BRFSS) 2012 data [29].

To describe the overall statewide distribution of relevant study variables, means and standard deviations or medians and ranges were generated for each state-level variable. Data were also described using histograms (see Additional file 1). Median values were used to describe meditation characteristics due to their non-normal distribution as evaluated by visual examination and the Shapiro-Wilk normality test. Cancer rates and health rankings exhibited normal distributions; therefore, mean values were used to describe these variables.

A correlation analysis between meditation variables and cancer rates or health rankings was conducted. Pearson correlation coefficients (ρ) and 2-sided p-values were calculated at the state level for each meditation variable and cancer incidence rate or health rank, separately. Data cleaning, frequency tables, and correlation analyses were performed with SAS® (version 9.3, SAS Institute, Cary, NC).

A geographic information system (GIS) (ArcMAP® software, version 10.1, ESRI, Redlands, CA) was used to overlay meditation characteristics and cancer incidence rates or health rankings by state to identify potential patterns of geographical overlap. Within the GIS, data were added to the polygon shapefile for the United States to allow for the symbolic display of two variables simultaneously. Cancer rates and health ranking data were displayed using graduated colors, in equal interval quartiles. The meditation data were displayed using circle symbols in graduated colors in natural breaks as defined by GIS because of its non-normal distribution.

## Results

Initially, there were 614 records in the United States-based MBSR teacher database at the time of data retrieval (September 2013). Duplicate records (N = 30) were identified (i.e., duplicate names in the same state, with some people having >2 records). For duplicate records, the maximum value for each meditation variable (hours teaching MBSR, years teaching MBSR, years of meditation practice) was extracted as representative of that person. Following this extraction process, the remaining duplicate records were deleted, leaving 571 records for analysis.

Table 1 presents the descriptive characteristics for state-level meditation characteristics, age-adjusted cancer incidence rates, and health rank variables. The median number of MBSR teachers per 100,000 population was 0.16, with a median of 76.58 hours teaching MBSR per 100,000 population, and 0.89 years teaching MBSR per 100,000 population (Table 1). As expected, female breast and prostate cancer had the highest age-adjusted mean incidence rates at 123.43/100,000 population and 144.53/100,000 population, respectively; the lowest incidence rate was for Hodgkin’s lymphoma (3.24/100,000 population; Table 1).

**Table 1:** **Descriptive characteristics for meditation, cancer, and health rank variables across all states (N = 50)**

**Variable**	**Median/mean**	**Range/SD**
Meditation characteristics*	***Median***	***Range***
Number of certified MBSR teachers	0.16	0 - 1.12
Hours teaching MBSR	76.58	0 - 604.73
Years teaching MBSR	0.89	0 - 6.23
Years of general meditation practice	2.51	0 - 18.70
Age-adjusted cancer incidence rates (2007–2011)*	***Mean***	***SD***
Colorectal	50.17	5.32
Female Breast	123.43	7.32
Prostate	144.53	16.84
Lung & bronchus	79.36	16.35
Melanoma	25.66	5.32
Liver	10.06	2.55
Non-Hodgkin’s lymphoma	23.07	2.10
Hodgkin’s lymphoma	3.24	0.53
Cervical	7.51	1.40
Health ranking data*	***Mean***	***SD***
Overall health	25.42	14.67
Mental health	24.69	14.56

MBSR: Mindfulness-based stress reduction; SD: standard deviation.

*Age-adjusted and per 100,000 population. Cancer data source: National Program of Cancer Registries: Incidence, WONDER online database. United States Department of Health and Human Services, Center for Disease Control and Prevention and National Cancer Institute: 2014.

Note: Cancer incidence data for 49 states and the District of Columbia were included; data for Nevada were unavailable because the cancer registry did not meet publication criteria for the years under consideration and was excluded from all analyses.

The results of the correlation analysis are presented in Table 2. Expectations were that ‘higher’ meditation characteristics would be inversely correlated with mean cancer incidence rates or health rankings (a lower rank indicates a healthier state). Negative correlations were detected between meditation characteristics and colorectal and cervical cancer, with weak to moderate (ρ: -0.495 - -0.264) correlation coefficients and statistically significant (p < 0.05) p-values for state-level meditation characteristics (Table 2). A negative correlation was detected between lung & bronchus cancer and years teaching MBSR only (ρ = −0.296, p = 0.04; Table 2). In general, no relationship was detected between melanoma, liver cancer, prostate cancer, or non-Hodgkin’s lymphoma and meditation characteristics (Table 2). Significant (p < 0.05) moderate positive correlations (ρ: 0.422 – 0.616; Table 2) were detected between Hodgkin’s lymphoma and female breast cancer in relation to meditation characteristics.

**Table 2:** **Pearson correlation coefficients between state-level meditation characteristics and state-level health rankings (N = 50)**

	Number of certified MBSR teachers*		Hours teaching MBSR*		Years teaching MBSR*		Years of general meditation practice*	
Age-adjusted cancer incidence rate*	**ρ**	**p-value**	**ρ**	**p-value**	**ρ**	**p-value**	**ρ**	**p-value**
Colorectal	−0.391	0.01	−0.312	0.03	−0.468	0.001	−0.406	0.004
Female breast	0.601	<0.0001	0.463	0.001	0.523	0.0001	0.616	<0.0001
Prostate	0.153	0.29	0.161	0.27	0.104	0.48	0.124	0.40
Lung & bronchus	−0.204	0.16	−0.163	0.26	−0.296	0.04	−0.216	0.14
Melanoma	0.178	0.22	0.209	0.15	0.282	0.05	0.217	0.13
Liver	0.269	0.06	0.222	0.13	0.273	0.06	0.218	0.13
NHL	0.212	0.14	0.264	0.07	0.213	0.14	0.255	0.08
HL	0.580	<0.0001	0.422	0.003	0.478	0.001	0.528	<0.0001
Cervical	−0.417	0.003	−0.264	0.07	−0.495	0.0003	−0.492	0.0003
***Health ranking data***	**ρ**	**p-value**	**ρ**	**p-value**	**ρ**	**p-value**	**ρ**	**p-value**
Overall health	−0.576	<0.0001	−0.460	0.001	−0.632	<0.0001	−0.617	<0.001
Mental health	−0.105	0.48	−0.183	0.21	−0.167	0.26	−0.152	0.30

MBSR: Mindfulness-Based Stressed Reduction; NHL: non-Hodgkin’s lymphoma; HL: Hodgkin’s lymphoma.

*Age-adjusted and per 100,000 population. Cancer data source: National Program of Cancer Registries: Incidence, WONDER online database. United States Department of Health and Human Services, Center for Disease Control and Prevention and National Cancer Institute: 2014.

N: Number of states (out of 51) included in analysis.

ρ: Two-tailed Pearson correlation coefficient.

Note: Cancer incidence data for 49 states and the District of Columbia were included; data for Nevada were unavailable because the cancer registry did not meet publication criteria for the years under consideration and was excluded from all analyses.

For health ranking data, all correlation coefficients were in the negative direction, in support of our hypothesis with meditation practices and number of teachers. Statistically significant correlations with moderate coefficients were detected for overall health rankings and all meditation characteristics, most strongly for total number of years teaching MBSR (ρ = −0.632; p < 0.0001; Table 2). Although not statistically significant, weak negative correlations between all meditation characteristics and mental health rankings were consistently detected (Table 2).

**Figure 1:**
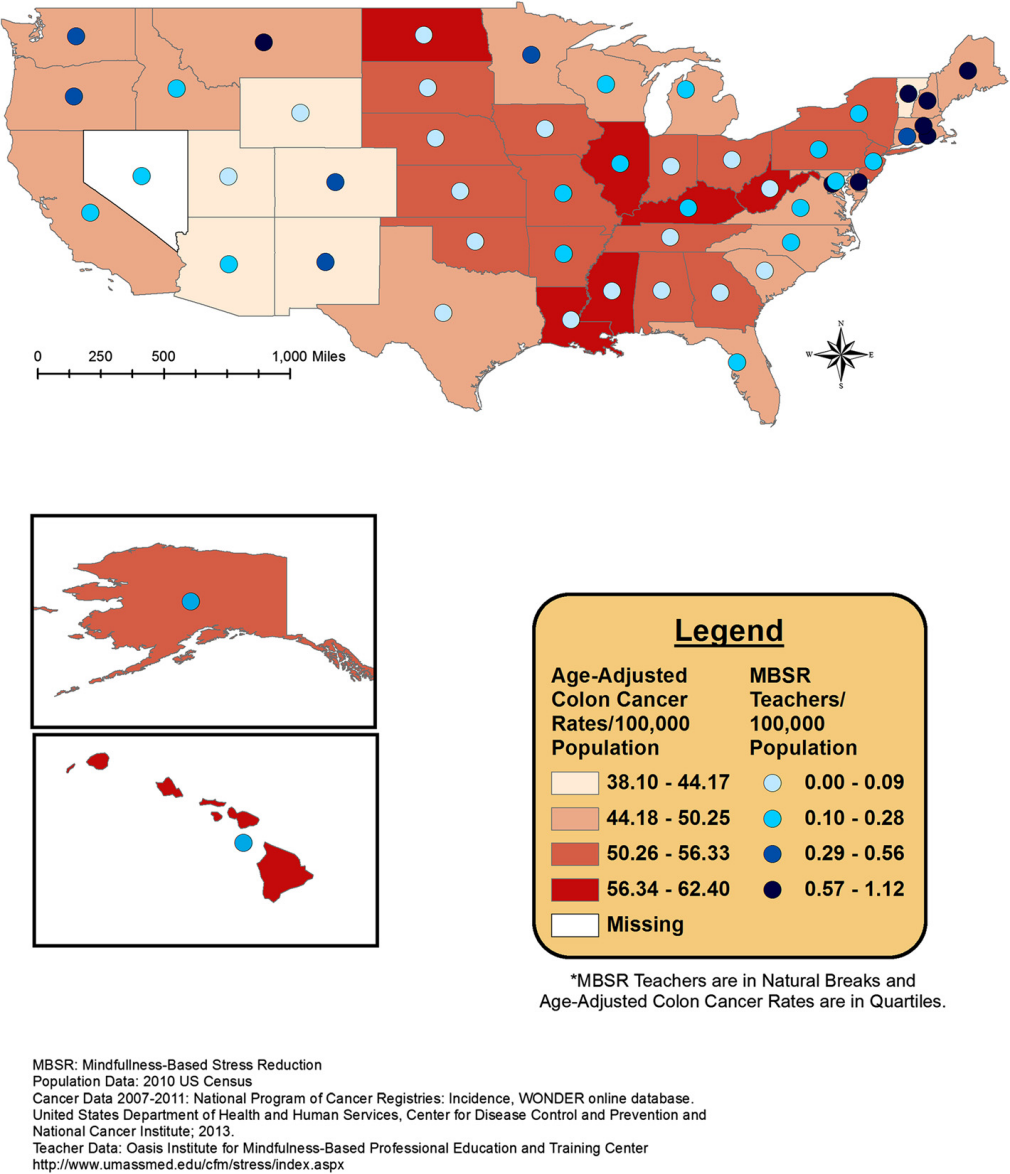
**Number of MBSR meditation teachers per 100,000 population and age-adjusted colorectal cancer incidence rates (2007–2011).**

**Figure 2:**
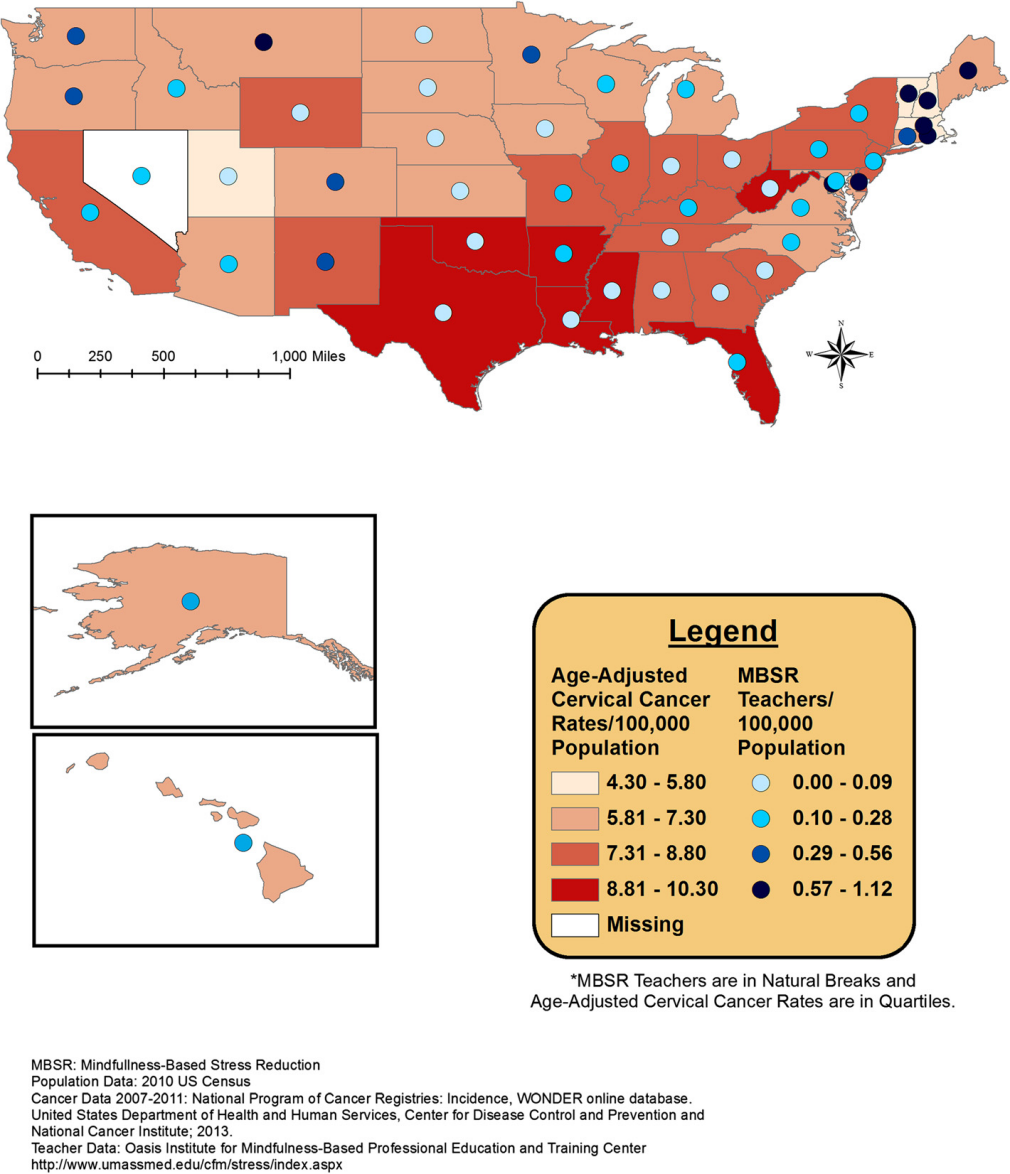
**Number of MBSR meditation teachers per 100,000 population and age-adjusted cervical cancer incidence rates (2007–2011).**

**Figure 3:**
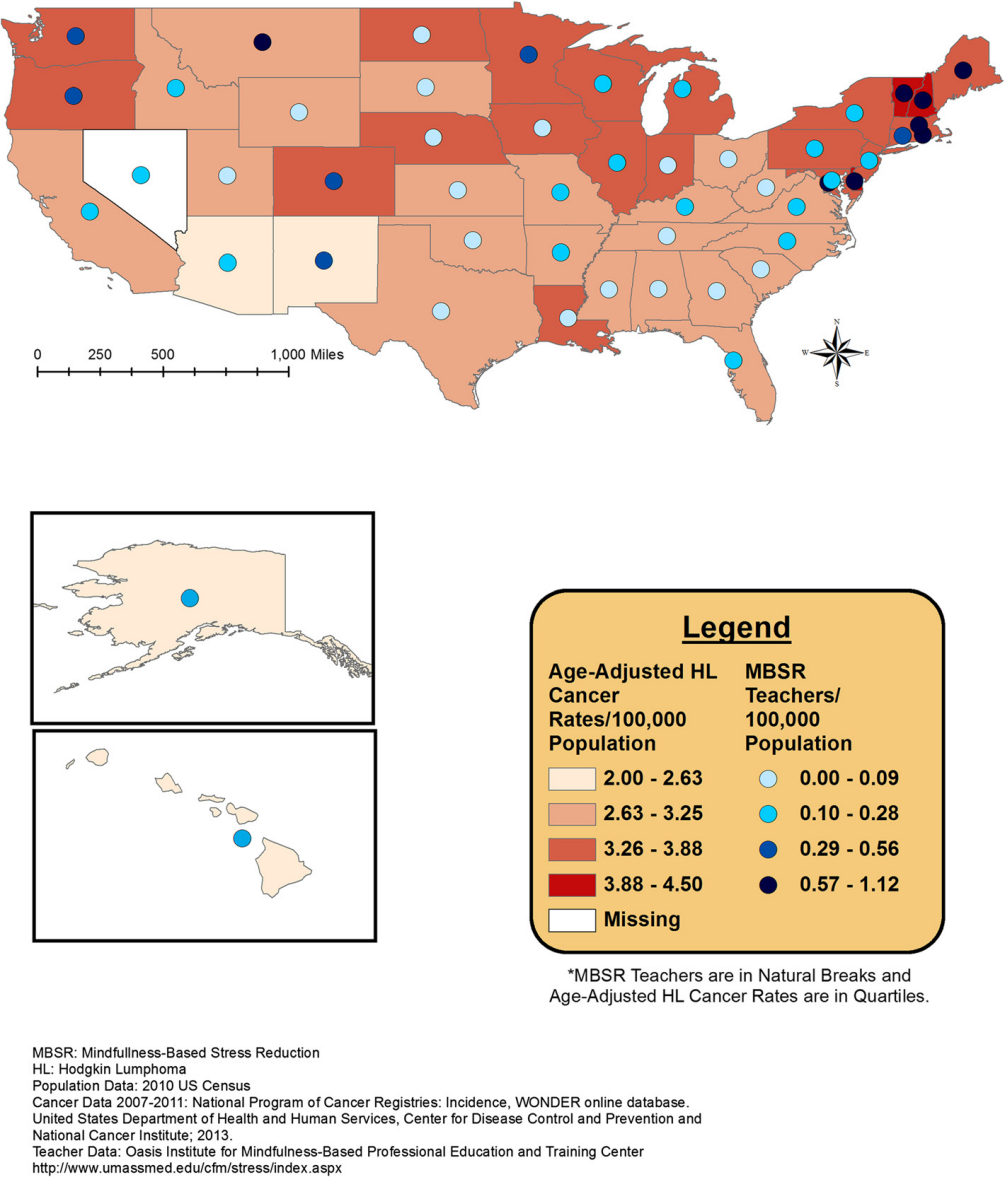
**Number of MBSR meditation teachers per 100,000 population and age-adjusted Hodgkin’s lymphoma incidence rates (2007–2011).**

**Figure 4:**
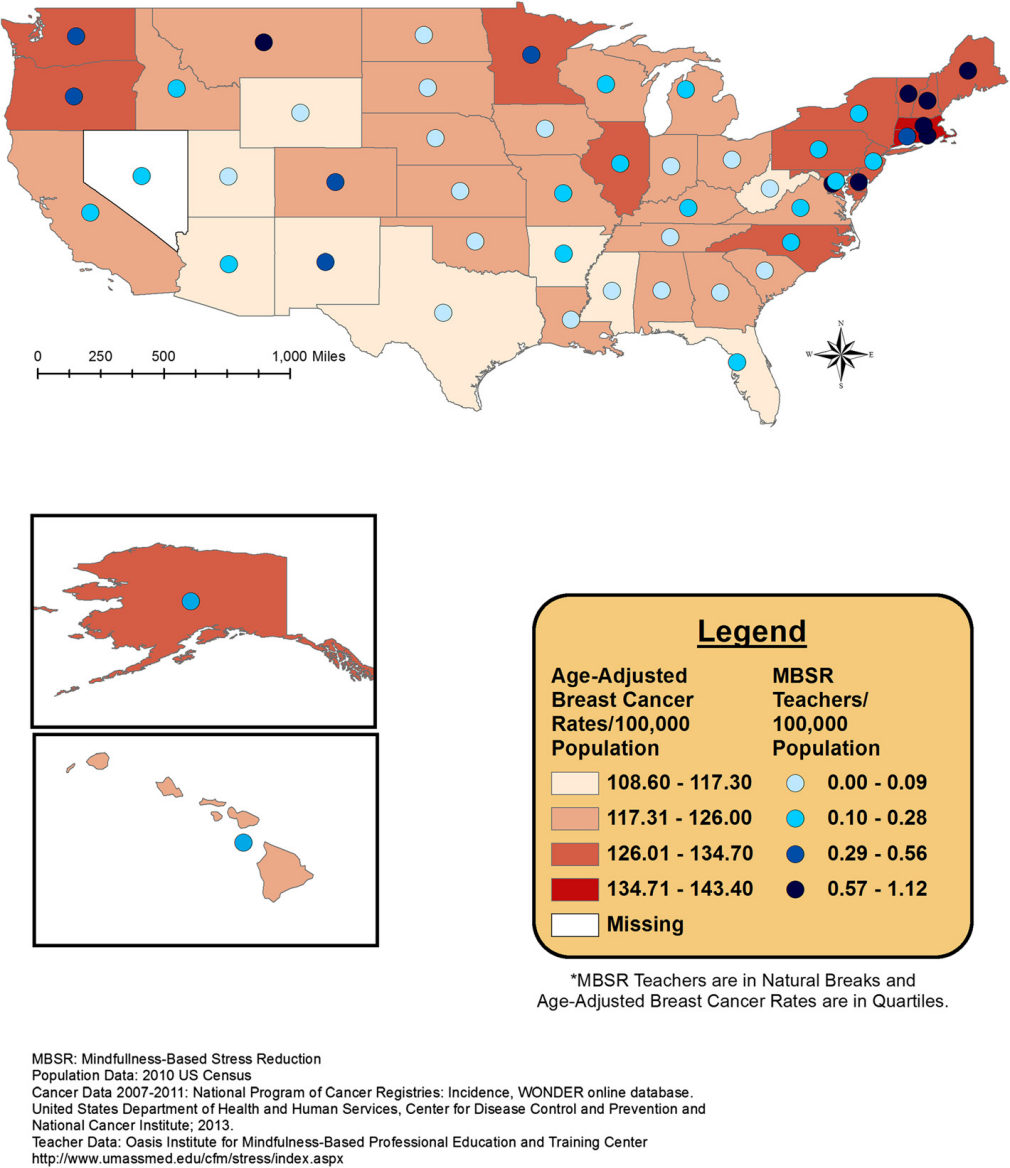
**Number of MBSR meditation teachers per 100,000 population and age-adjusted female breast cancer incidence rates (2007–2011).**

**Figure 5:**
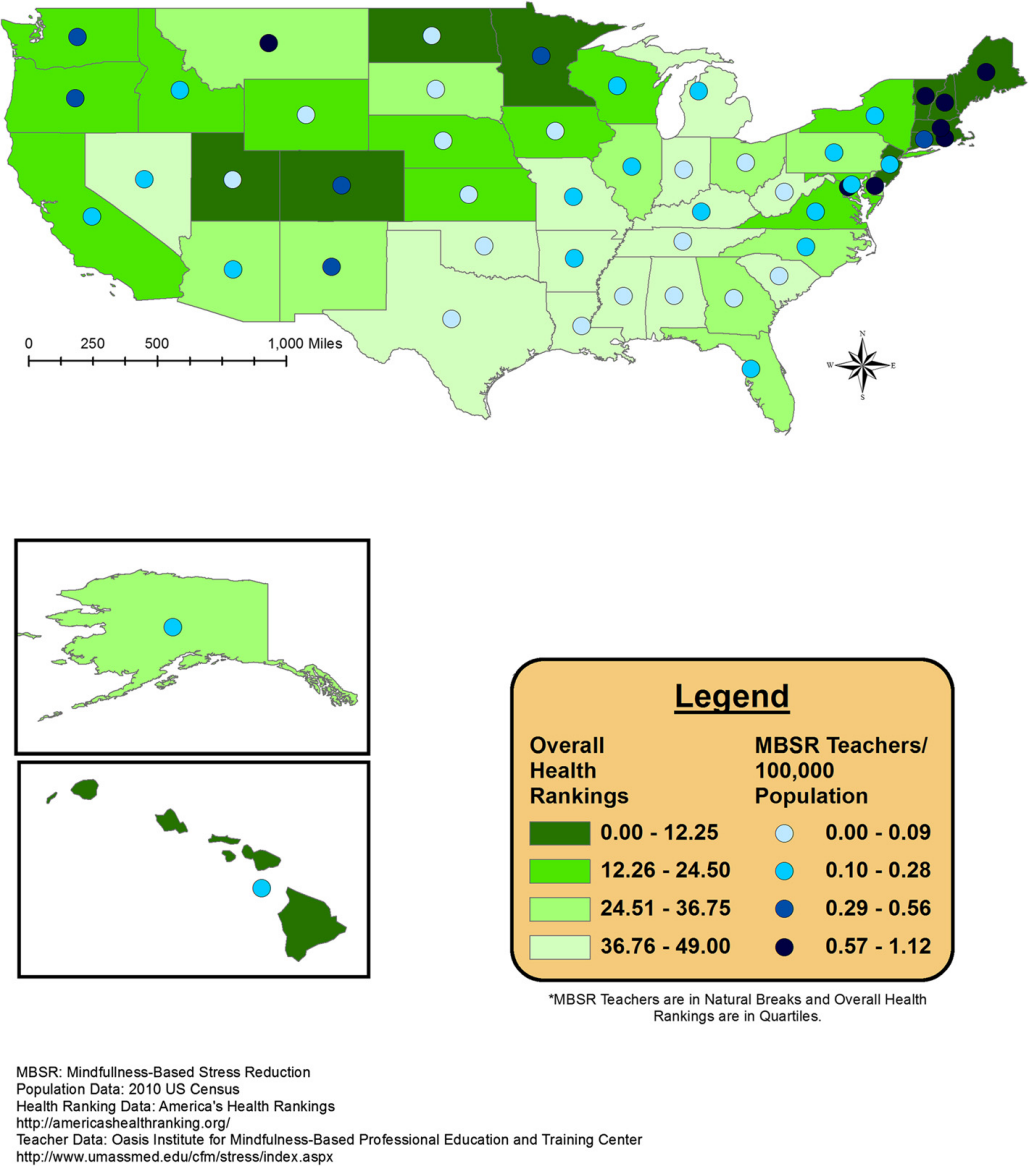
**Number of MBSR meditation teachers per 100,000 population and overall state health rankings.**

Overlaid geographic patterns of number of certified MBSR teachers and cancer incidence or health rank data are displayed at the state level in Figures 1, 2, 3, and 4. All four meditation characteristics were strongly positively correlated (Spearman correlation coefficients range: 0.84 – 0.98, p < 0.05), therefore, number of certified MBSR teachers was chosen as representative of potential geographic patterns with various health characteristics. More meditation teachers were located in the northeast and western United States. In the remainder of the United States, Montana fell in the highest category (0.57-1.12 MBSR teachers/100,000 population) and Washington, Oregon, Colorado, New Mexico, and Minnesota fell in the second highest category (0.29-0.56 MBSR teachers/100,000 population) (see gradated blue circles in Figures 1, 2, 3, 4, and 5). Similar patterns were observed, as expected due to strong positive correlations, for the other meditation characteristics (data not shown).

Figure 1 displays the age-adjusted colorectal cancer incidence rates and the total number of MBSR teachers. Figure 2 displays the age-adjusted cervical cancer incidence rates and the total number of MBSR teachers. Higher colorectal and cervical cancer incidence rates were observed in the eastern portion of the United States, with a group of states with higher age-adjusted cervical cancer incidence rates around Texas; meditation characteristics displayed the most prominence in the north-eastern United States (Figures 1 and 2). The geographic overlap for total number of MBSR teachers and age-adjusted Hodgkin’s lymphoma and female breast cancer are displayed in Figures 3 and 4, respectively. For both of these cancers, states in the northeastern United States and Washington, Oregon, and Minnesota had a combination of higher cancer incidence rates and a higher total number of MBSR teachers.

Overall health rankings are displayed with the number of MBSR teachers in Figure 5. The north-eastern United States represents an area with overall state health rankings in the lowest (healthiest) quartile (0–12.25) and total number of meditation teachers in the highest category (0.57-1.12; Figure 5). Maps for cancers that showed no correlation with meditation characteristics (liver, non-Hodgkin’s lymphoma, prostate, melanoma, and lung & bronchus cancer) and mental health data are available as Additional files 2, 3, 4, 5, 6 and 7 for the interested reader.

## Discussion

This paper provides a broad, state-level description of meditation characteristics and health data using descriptive statistics, correlation analysis, and spatial overlay conducted with GIS. As such, it represents a previously unexplored area of research using a unique descriptive methodological approach to discerning ecologic state-level patterns. Our analysis suggests state-level correlations between meditation characteristics and colorectal and cervical cancer incidence, with states with more meditation (e.g., more MBSR teachers per population) correlated with a decreased cancer incidence. A similar relationship was suggested for years teaching MBSR and lung & bronchus cancer incidence, although results were less conclusive.

Long-term meditators provide a unique opportunity to investigate virtually unexplored areas of meditation such as its role in chronic disease prevention via its impact on stress reduction. Chronic stress may be related to cancer through a variety of biobehavioral pathways [6]. Stress affects inflammation [9], which has been most strongly associated with colon cancer [7]. For example, inflammatory bowel disease and other sporadic and heritable forms of colon cancer are linked directly with inflammation and more than 20% of patients with inflammatory bowel disease develop a specific subtype of colon cancer, colitis-associated cancer [30]. We were unable to identify specific colon cancer subtypes in these analyses. In addition, analysis of one large prospective study from Japan documented a weak positive association between perceived stress and colon cancer mortality in women [31]. Other studies, however, have reported opposing results [32].

Stress also lowers the body’s response to viral challenge. Emotional stress can trigger reactivation of latent viruses [2]. This immune system dysregulation has been linked with increased incidence of non-Hodgkin’s lymphoma and immunogenic tumors such as lymphomas, cervical cancer, liver cancer, and basal cell carcinoma [7,8]. Specific subtypes of the human papillomavirus (HPV) have been linked to an increased risk of cervical cancer [33]. Recent studies have evaluated the role of cell-mediated immune responses to HPV and determined that higher levels of immunosuppression are correlated with an increased risk of cervical cancer. In a sample of 74 women, Fang (2008) found an association between higher levels of perceived stress with a non-response of T-cells to HPV16, the most common subtype linked to cervical cancer [33]. These findings support the notion that stress reduction may be important in colorectal and cervical cancer incidence and warrants further investigation.

The relationship detected between state-level number of MBSR teachers and colorectal or cervical cancer incidence rates may be confounded by socioeconomic status (SES), which was not adjusted for in this descriptive examination. State-level median household income and the number of MBSR teachers per population were moderately positively correlated in our data (Spearman correlation coefficient = 0.45, p = 0.001). In addition, we recently collected primary data on this group of long-term MBSR meditators and characterized 62% of the 84 meditators on whom complete data were available as having a Master’s degree or higher (data not shown), indicative of a high SES. Both colorectal and cervical cancers are potentially preventable through the detection and treatment of pre-cancerous legions (i.e., polyps, cervical intraepithelial neoplasia [CIN]) through screening. Individuals with a higher SES are likely to have a better knowledge about the importance of screening and increased access to appropriate cancer screening facilities. If proper screening recommendations are followed, and to a greater extent among individuals with a high SES, colorectal and cervical cancers may be detected in a pre-cancerous stage and not included as incident cancers in the registry. Our findings may reflect this underlying mechanism of action. Detection of pre-cancerous legions for other cancer sites (e.g., breast, prostate) is not well understood and only currently being developed [34].

No relationship was detected between meditation characteristics and non-Hodgkin’s lymphoma, melanoma, prostate, or liver cancer. While a negative correlation in support of our hypothesis was detected between colorectal and cervical cancer and meditation characteristics, moderate positive correlations were detected between meditation characteristics and Hodgkin’s lymphoma and female breast cancer. The ecologic, hypothesis-generating nature of this study is not designed to detect fine-level patterns and could be responsible for these unexpected results. These findings may be explained by variations SES, which were not accounted for in this ecologic description. A higher SES has been strongly associated with an increased risk of breast cancer [35] and Hodgkin’s lymphoma [36] and may have confounded these results, especially given that this population of meditators is of high SES, as discussed above. Although the risk of Hodgkin’s lymphoma increases with increasing SES, studies have suggested an opposite pattern with incidence of non-Hodgkin’s lymphoma, which was also reflected by our data. Two recent studies found an inverse association (odds ratio [OR] = 0.86; 95% confidence interval: 0.79-0.94) between SES and non-Hodgkin’s lymphoma overall [37] and per increasing tertile of SES [38]. Future studies should carefully control for SES factors to further evaluate these associations.

Correlations between meditation characteristics were observed with overall health rankings at the state level, with states with more meditation correlated with an overall healthier state ranking. No relationship was detected with mental health rankings. The overall health ranking is a weighted composite of length of life (years of potential life lost before age 75) and quality of life (poor or fair health; poor physical health days; poor mental health days). Although we expected to see a stronger relationship between meditation and mental health characteristics specifically, the overall health ranking may capture more complex health-related patterns. We were unable to identify any geography-specific stress data, and therefore, utilized statewide overall and mental health data as a surrogate marker for stress. The mental health data were compiled as the number of days in the previous 30 days that a person could not perform work or household tasks due to mental illness. Although this metric provided a “general indication of health-related quality of life, mental distress, and the burden of more serious mental illnesses [39],” it does not directly measure psychological stressors. Individual-level data on mental health conditions is necessary to confirm or refute these findings.

The skills acquired in an MBSR program have been shown to reduce psychological distress and promote well-being. In 2010, Merkes identified an overall increase in quality of life, coping strategies, and general health outcomes in 15 studies utilizing MBSR programs for adults with chronic diseases. All eight studies that measured anxiety found a decrease in anxiety levels, the majority of which were statistically significant reductions [21]. Even shortened versions of the standard eight-week course have proven effective in stress reduction. Bergen-Cico et al. demonstrated an increase in mindfulness and self-compassion in a nonclinical population of college students after a 5-week MBSR program [40]. However, the research on the correlation between duration and frequency of meditation and health benefits is scarce [25], with few studies to date examining the impact of long-term meditation [11,26]. Long-term meditators present potential opportunities to learn about this relationship. Massion et al.’s study identified a relationship between longer-term meditators and increased melatonin levels [11]. Brand et al.’s recent study examined the impact of long-term meditation (average: 264 months) on stress among a group of 20 subjects and found a reduction in cortisol levels irrespective of age [26]. The literature on mindfulness-based approaches for cancer patients has focused almost entirely on coping strategies and quality of life measures [16,17,41]. A recent study found that breast cancer survivors participating in a mindfulness-based cancer recovery program maintained their telomere length, which has been associated with improved disease prognosis [42]. Therefore, there is ample scope for studies examining meditation and its prevention value.

This ecologic examination identified some state-level geographic overlap between higher numbers of meditation teachers and more time spent practicing and better health outcomes in the northeastern United States and specific areas within the midwest portion of the United States. Although these patterns may be an artifact of varying characteristics of residents in these geographic regions, such as health care access or SES, these initial results indicate that exploring this research topic using alternative strategies and methods may yield important results. In addition, adults residing in the eastern portion of the United States are more stressed and have experienced increasing stressors over time as compared to the remainder of the nation [1], making focused research on this topic a priority. Throughout all geographic areas within the United States, adults are turning to exercise, healthy eating, watching television, reading, and listening to music to manage their stress levels [1]. Though these mechanisms may provide a useful outlet for stress reduction, meditation represents an easily implemented, cost efficient, and scientifically supported method for the reduction of stress.

The MBSR database is based on self-report and is continuously updated as teachers change their status. For example, since our initial access of this database in September 2013, approximately 3–5 additions were made to the database and 2–3 individuals were no longer included, yielding a net increase of about 1–2 additions every month. In addition, no data on the mobility of meditation teachers (e.g., the number of years or hours of MBSR teaching in each state) were available, which limited the scope of our analysis. Future studies may assess the temporal change in numbers of teachers, characteristics of teachers, and/or geographic distribution of teachers to capture patterns in meditation use and education throughout the nation.

This study was limited to individuals with training or certification in the MBSR program. The use of several mind-body therapies has increased in recent years among United States adults, with the prevalence of reported past-year usage of meditation, yoga and deep breathing exercises all significantly increasing from 2002 to 2007. In particular, the prevalence of reported meditation usage within the last 12 months among United States adults rose from 7.6% in 2002 to 9.4% in 2007 [43]. Future studies should assess the impact on stress and cancer outcomes stratified by meditation type or mind-body technique.

This descriptive state-level analysis was not intended to evaluate causal inferences between meditation characteristics and health outcomes. Caution should be made in interpreting these results, as ecologic fallacy may be at play. No regression models were evaluated due to the ecologic nature of this analysis and the lack of data on potentially confounding variables, such as socioeconomic status, population density, and race, limits the application of these study results. Based on these initial results, additional analyses including spatial regression models incorporating these types of variables, as well as the collection of individual-level meditation and health data, especially as it relates to mental health, stress, and certain cancer outcomes, is warranted.

## Conclusions

This descriptive study represents a first step in an important line of research exploring the potential relationship between long-term meditation, stress, and cancer incidence. Despite its limitations, this study is an important addition to the literature on meditation and health, as it is the first to examine the relationship between meditation and cancer from a geographic perspective. It serves as an important starting point for additional studies examining meditation practices and health outcomes, particularly as they relate to stress reduction and colorectal and cervical cancer incidence. In addition, this study identified certain geographic areas, such as the northeastern United States, where attention should be focused. Additional studies are underway in our research group to collect detailed individual-level meditation and stress information among this group of long-term meditators, which will provide a vital supplement to this state-level description.

## Additional files

Additional file 1:
**Variable Histograms.** Histograms of meditation, cancer, and health variables. Additional file 2:
**Number of MBSR meditation teachers per 100,000 population and age-adjusted liver cancer incidence rates (2007–2011).**
Additional file 3:
**Number of MBSR meditation teachers per 100,000 population and age-adjusted non-Hodgkin’s lymphoma incidence rates (2007–2011).**
Additional file 4:
**Number of MBSR meditation teachers per 100,000 population and age-adjusted prostate cancer incidence rates (2007–2011).**
Additional file 5:
**Number of MBSR meditation teachers per 100,000 population and age-adjusted melanoma incidence rates (2007–2011).**
Additional file 6:
**Number of MBSR meditation teachers per 100,000 population and age-adjusted lung & bronchus cancer incidence rates (2007–2011).**
Additional file 7:
**Number of MBSR meditation teachers per 100,000 population and state mental health rankings.**

## Abbreviations

CIN: Central intraepithelial neoplasia
HPV: Human papillomavirus
BRFSS: Behavioral risk factor surveillance system
GIS: Geographic information system
HPA: Hypothalamic-pituitary-adrenal axis
MBSR: Mindfulness-based stress reduction
SES: Socioeconomic status
