# Supplementary material for: Mindfulness-based stress reduction teachers, practice characteristics, cancer incidence, and health: a nationwide ecological description
Source: BMC Complement Altern Med. 2015 Feb 14;15:24. doi: 10.1186/s12906-015-0545-3 (PMC4342874; doi:10.1186/s12906-015-0545-3)

**Histograms of Age-Adjusted Specific Cancer Incidence Rates**


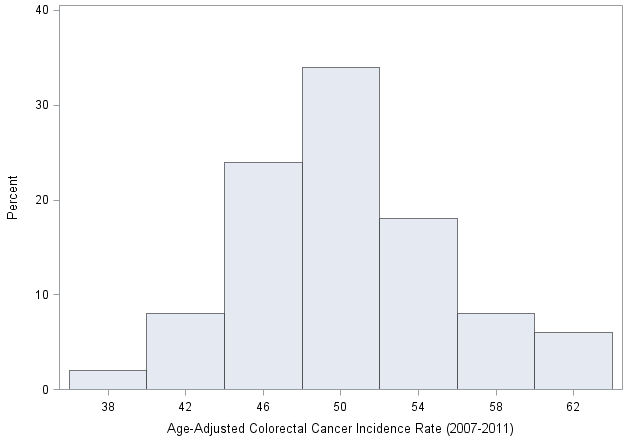

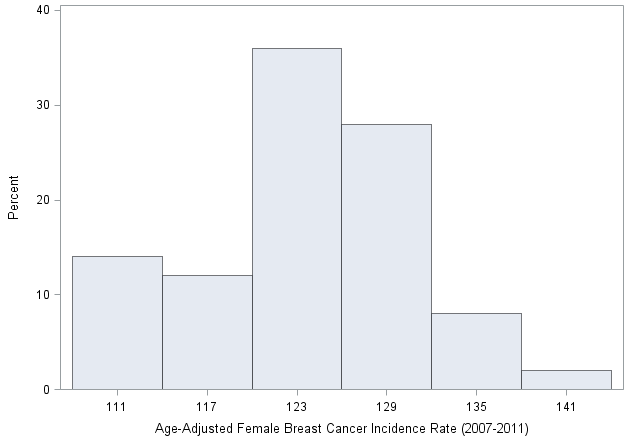


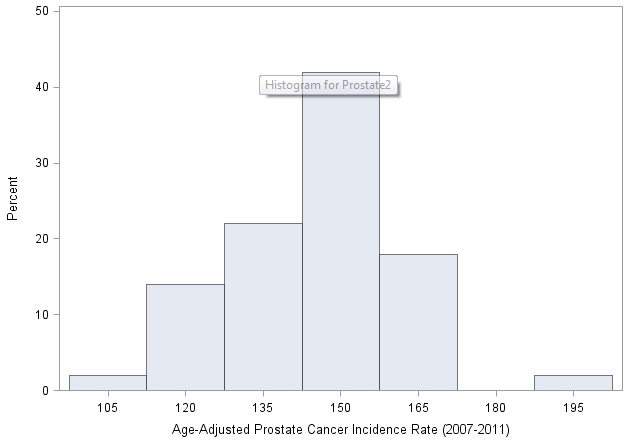

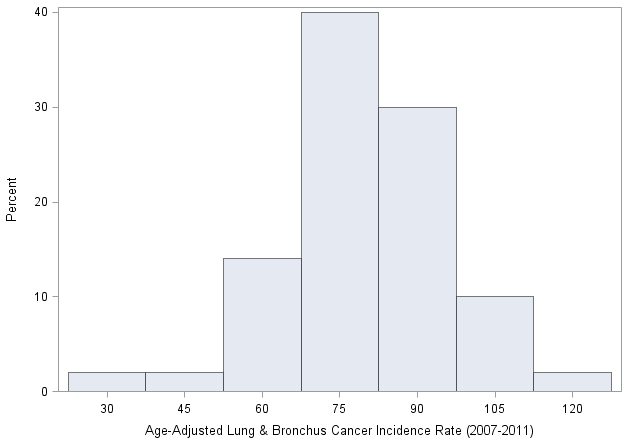


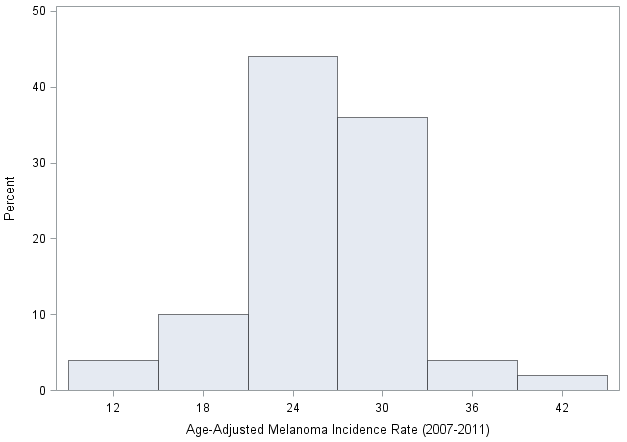

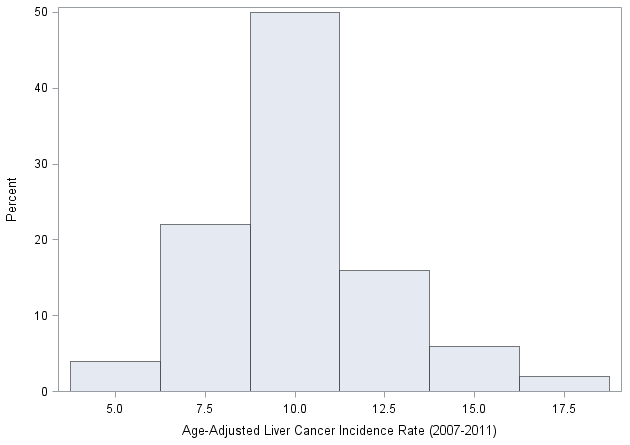


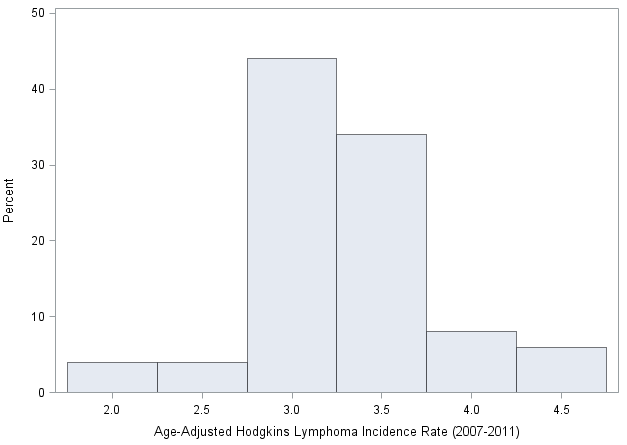

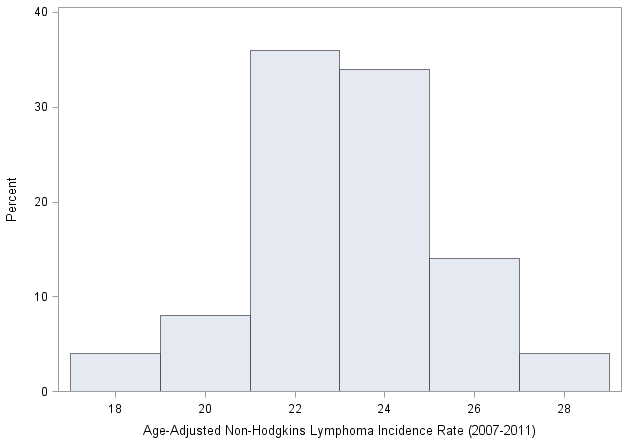


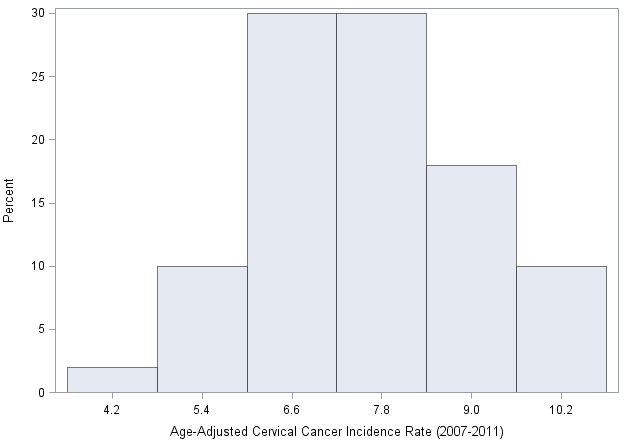


**Histograms of Meditation Characteristics**


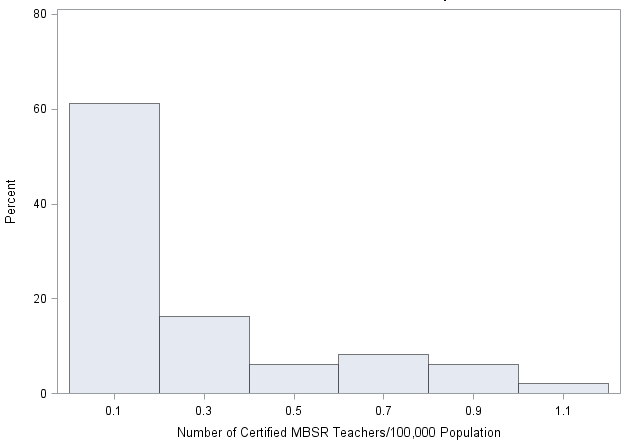

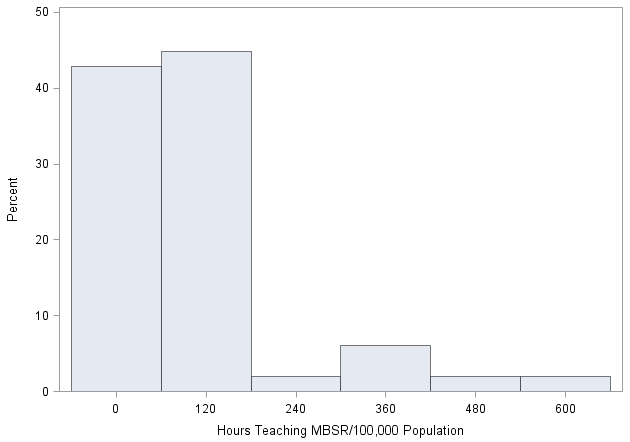


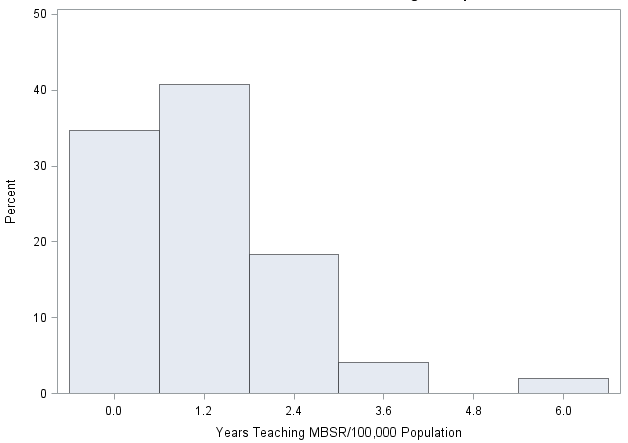

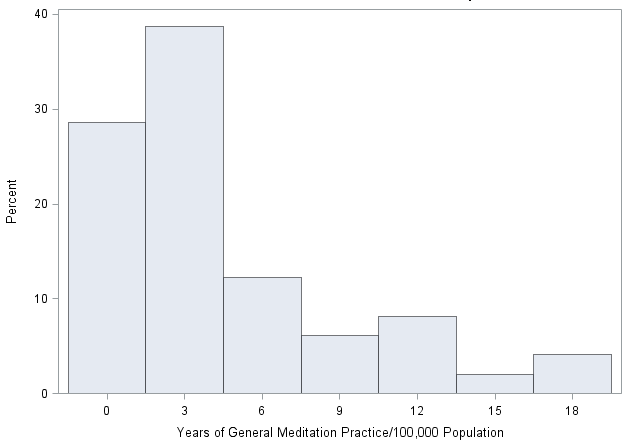


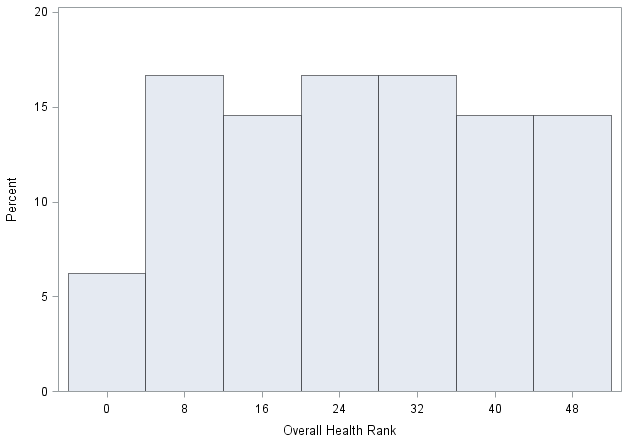

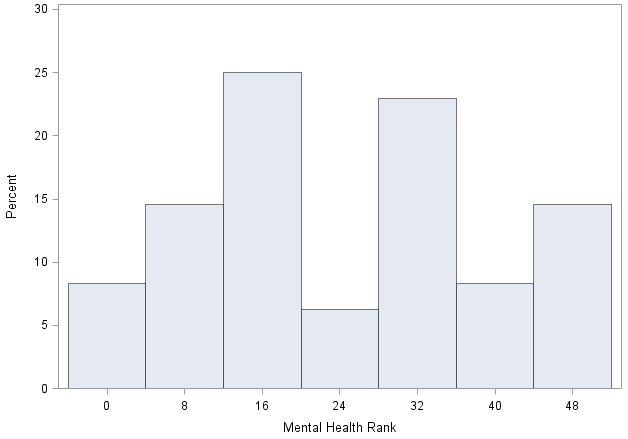

Supplement: Additional file 1: — Variable Histograms. Histograms of meditation, cancer, and health variables. [file 12906_2015_545_MOESM1_ESM.docx]
