# Supplementary figures and images for: Mindfulness-based stress reduction teachers, practice characteristics, cancer incidence, and health: a nationwide ecological description
Source: BMC Complement Altern Med. 2015 Feb 14;15:24. doi: 10.1186/s12906-015-0545-3 (PMC4342874; doi:10.1186/s12906-015-0545-3)

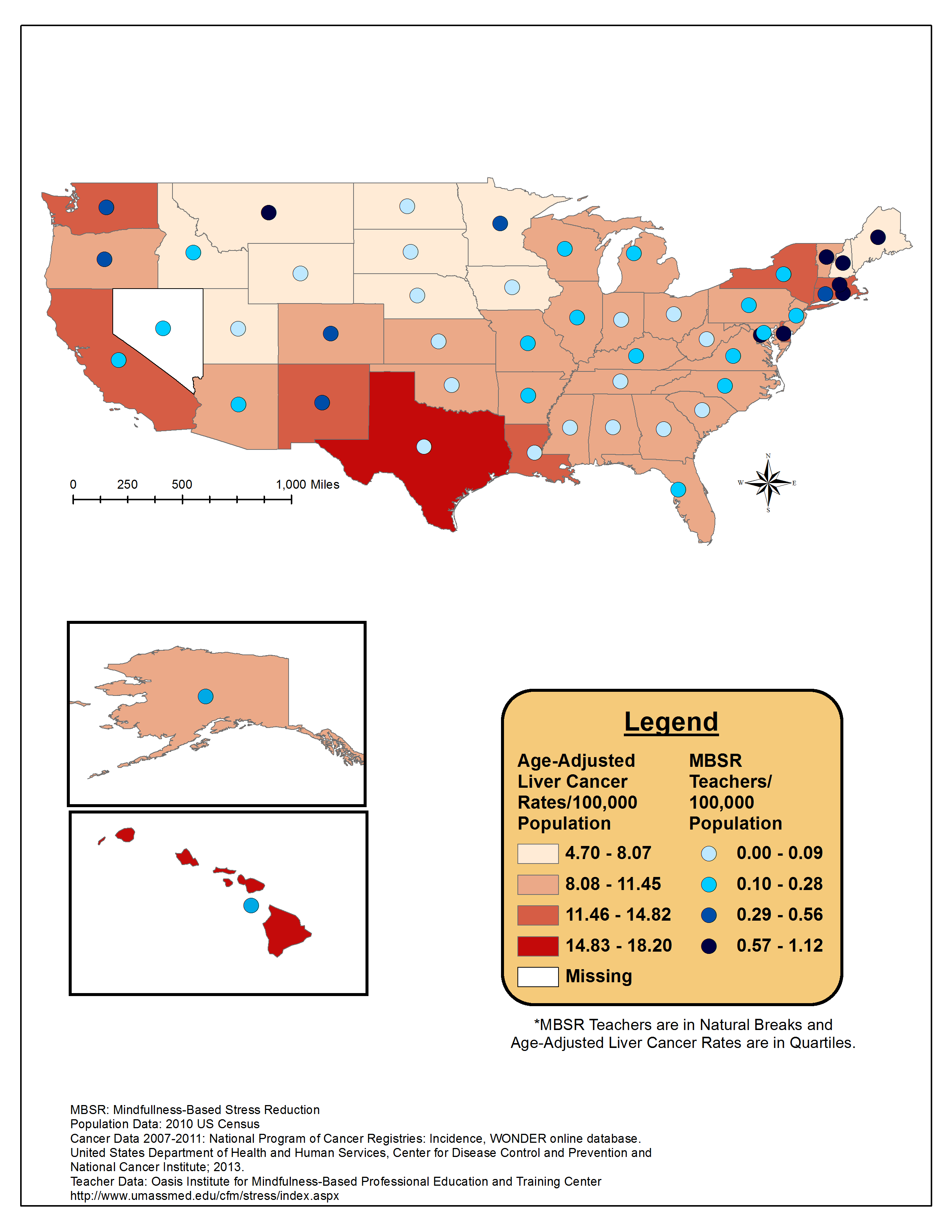

Supplement: Additional file 2: — Number of MBSR meditation teachers per 100,000 population and age-adjusted liver cancer incidence rates (2007–2011). [file 12906_2015_545_MOESM2_ESM.tiff]

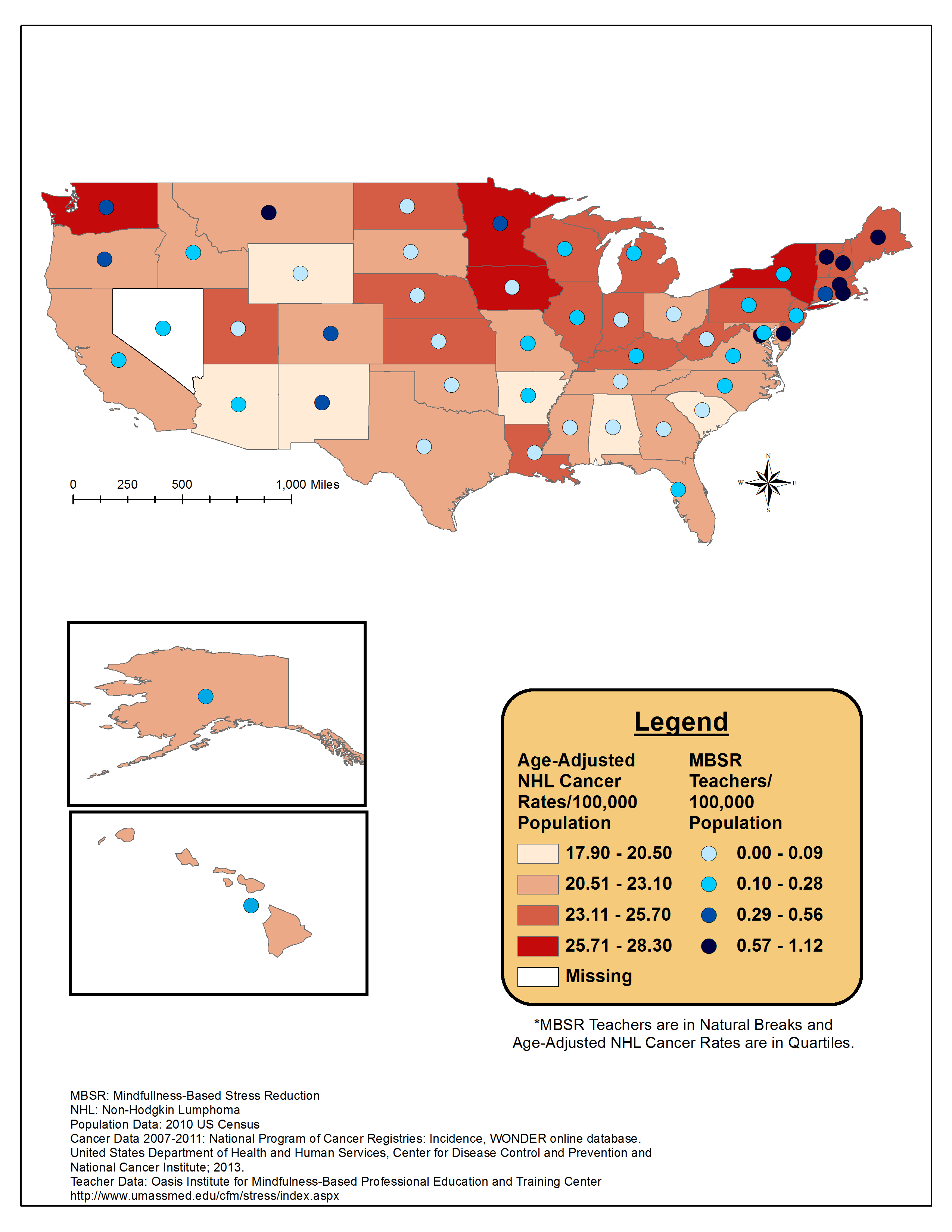

Supplement: Additional file 3: — Number of MBSR meditation teachers per 100,000 population and age-adjusted non-Hodgkin’s lymphoma incidence rates (2007–2011). [file 12906_2015_545_MOESM3_ESM.tiff]

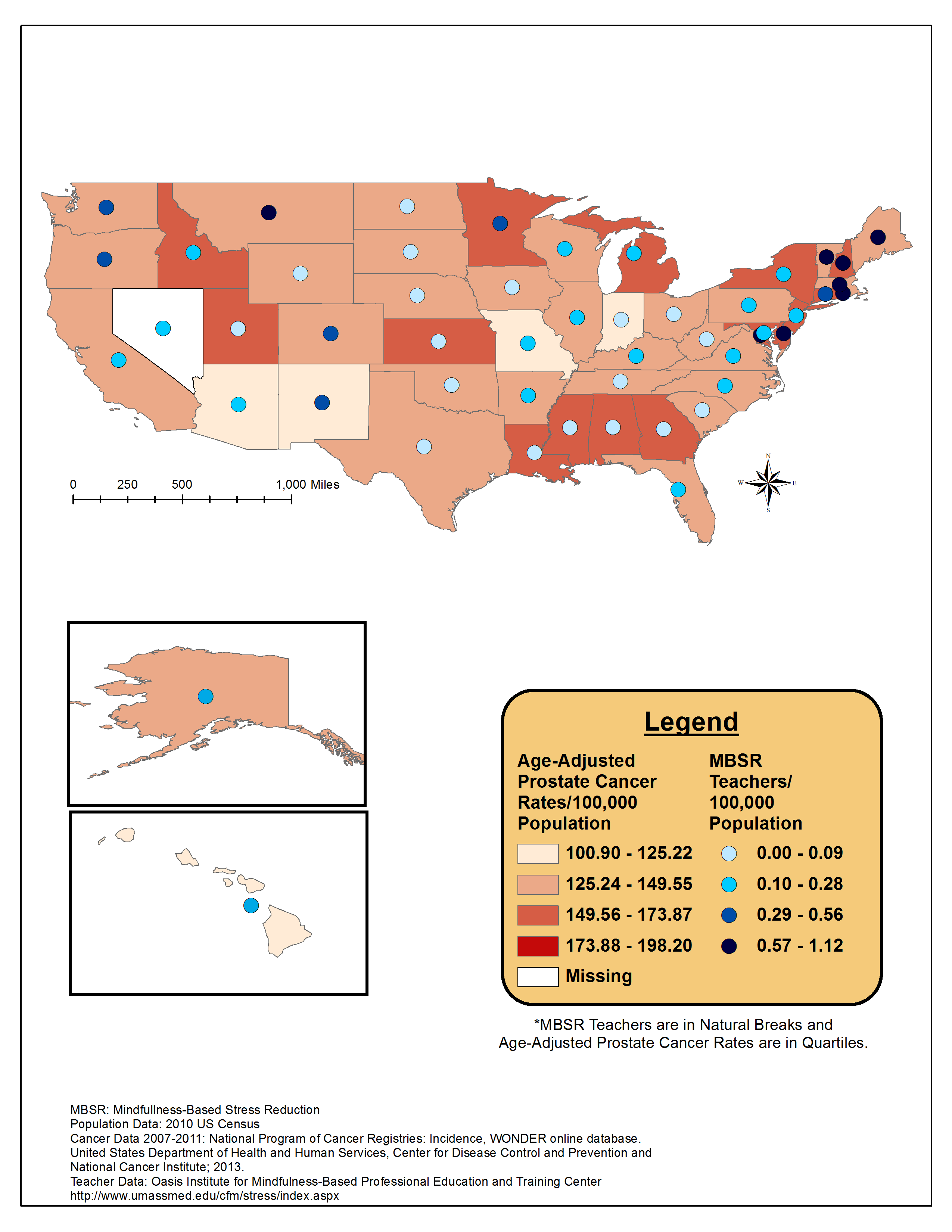

Supplement: Additional file 4: — Number of MBSR meditation teachers per 100,000 population and age-adjusted prostate cancer incidence rates (2007–2011). [file 12906_2015_545_MOESM4_ESM.tiff]

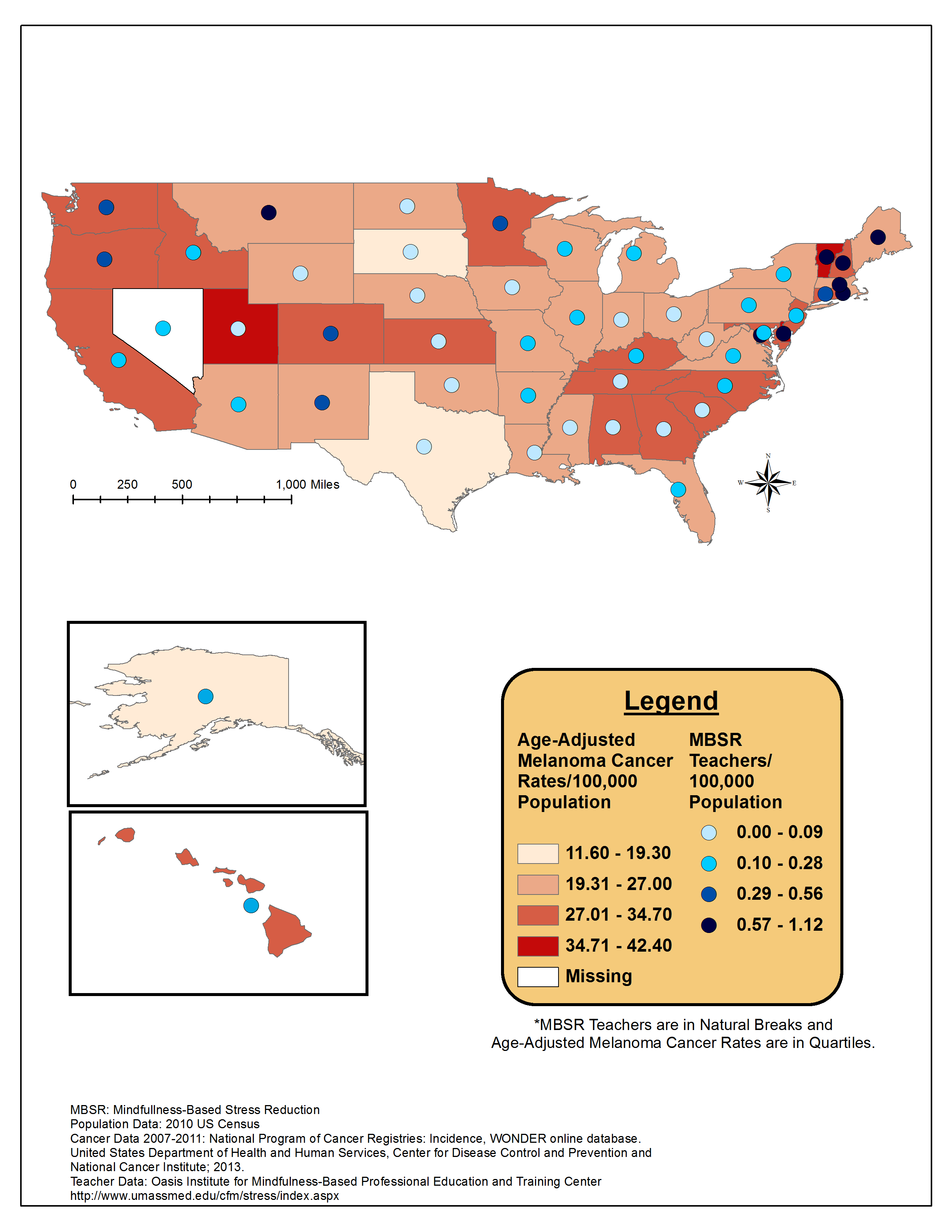

Supplement: Additional file 5: — Number of MBSR meditation teachers per 100,000 population and age-adjusted melanoma incidence rates (2007–2011). [file 12906_2015_545_MOESM5_ESM.tiff]

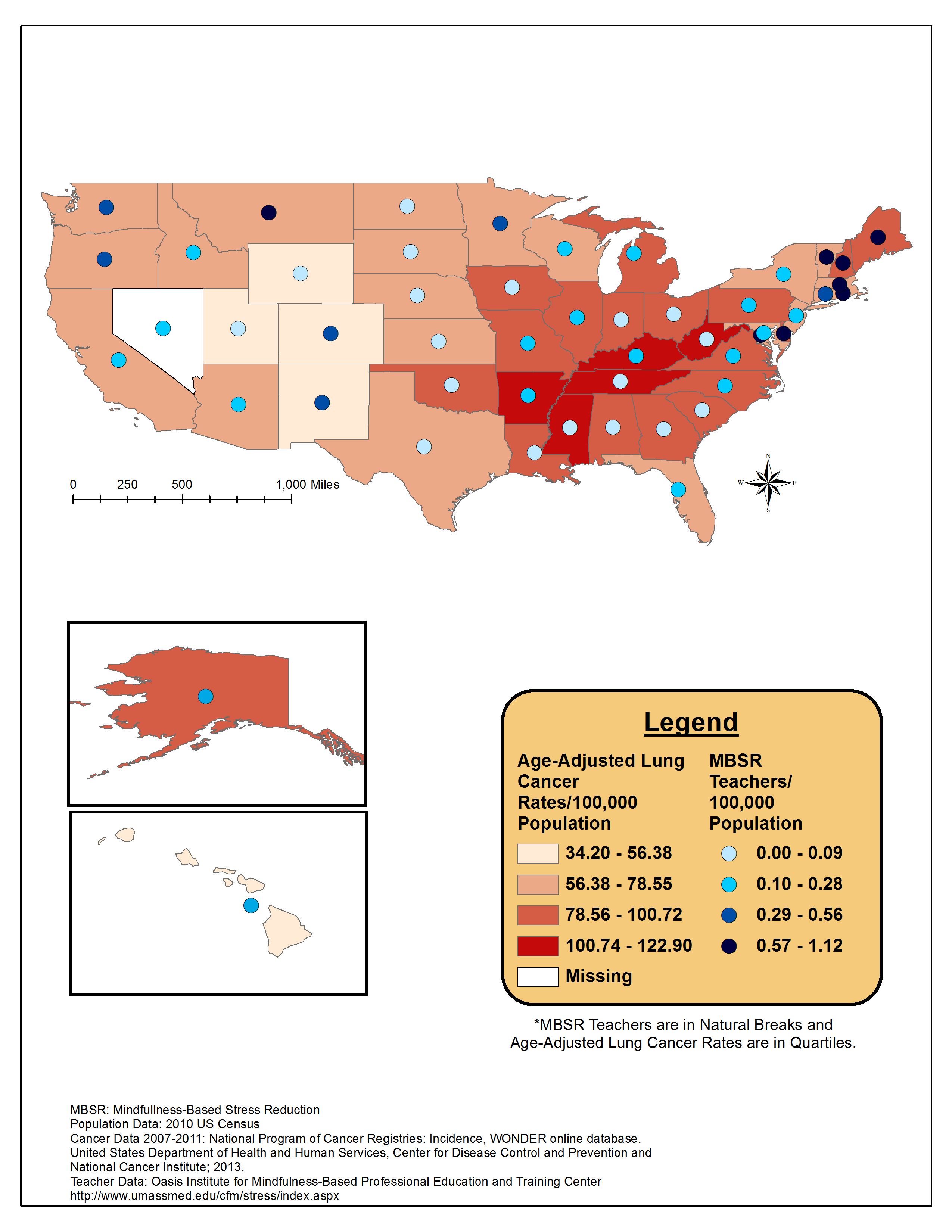

Supplement: Additional file 6: — Number of MBSR meditation teachers per 100,000 population and age-adjusted lung & bronchus cancer incidence rates (2007–2011). [file 12906_2015_545_MOESM6_ESM.tiff]

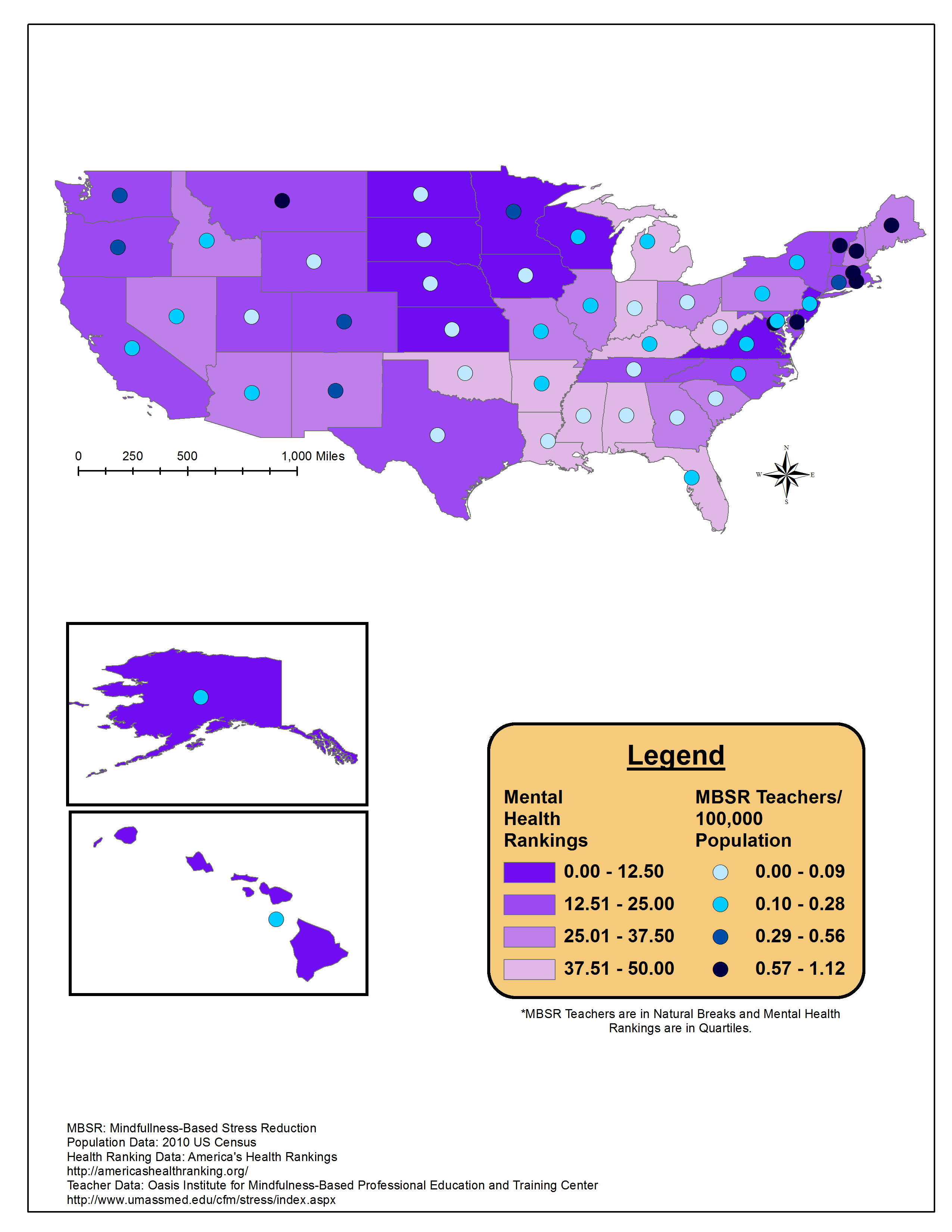

Supplement: Additional file 7: — Number of MBSR meditation teachers per 100,000 population and state mental health rankings. [file 12906_2015_545_MOESM7_ESM.tiff]
